# Circulating trimethylamine *N*‐oxide levels do not predict 10‐year survival in patients with or without coronary heart disease

**DOI:** 10.1111/joim.13550

**Published:** 2022-08-09

**Authors:** Espen Ø. Bjørnestad, Indu Dhar, Gard F. T. Svingen, Eva R. Pedersen, Stein Ørn, Mads M. Svenningsson, Grethe S. Tell, Per M. Ueland, Gerhard Sulo, Reijo Laaksonen, Ottar Nygård

**Affiliations:** ^1^ Department of Cardiology Stavanger University Hospital Stavanger Norway; ^2^ Mohn Nutrition Research Laboratory Department of Clinical Science University of Bergen Bergen Norway; ^3^ Department of Cardiology Haukeland University Hospital Bergen Norway; ^4^ Department of Clinical Science University of Bergen Bergen Norway; ^5^ Department of Global Public Health and Primary Care University of Bergen Bergen Norway; ^6^ Centre for Disease Burden Norwegian Institute of Public Health Bergen Norway; ^7^ Finnish Cardiovascular Research Center University of Tampere Tampere Finland

**Keywords:** cardiovascular risk factors, coronary artery disease, mortality, prevention, trimethylamine *N*‐oxide

## Abstract

**Background:**

Trimethylamine *N*‐oxide (TMAO) is an amine oxide generated by gut microbial metabolism. TMAO may contribute to atherothrombosis and systemic inflammation. However, the prognostic value of circulating TMAO for risk stratification is uncertain.

**Methods:**

We assessed prospective relationships of plasma TMAO with long‐term risk of all‐cause, cardiovascular (CV), and non‐CV mortality in the Western Norway Coronary Angiography Cohort (WECAC; 4132 patients with suspected coronary artery disease) and the Hordaland Health Study (HUSK; 6393 community‐based subjects). Risk associations were examined using Cox regression analyses.

**Results:**

Mean follow‐up was 9.8 and 10.5 years in WECAC and HUSK, respectively. Following adjustments for established CV risk factors and indices of renal function in WECAC, the hazard ratios (HRs) (95% confidence intervals [CIs]) per one standard deviation increase in log‐transformed plasma TMAO were 1.04 (0.97–1.12), 1.06 (0.95–1.18), and 1.03 (0.93–1.13) for all‐cause, CV, and non‐CV mortality, respectively. Essentially similar results were obtained in patients with angiographically significant coronary artery disease and patients with reduced left ventricular ejection fraction. Corresponding HRs (95% CIs) in the HUSK cohort were 1.03 (0.96–1.10), 1.01 (0.89–1.13), and 1.03 (0.95–1.12) for all‐cause‐, CV, and non‐CV mortality, respectively.

**Conclusions:**

Circulating TMAO did not predict long‐term all‐cause, CV, or non‐CV mortality in patients with coronary heart disease or in community‐based adults. This large study does not support a role of TMAO for patient risk stratification in primary or secondary prevention.

## Graphical abstract

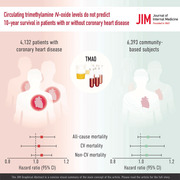

## Introduction

Circulating gut microbiota–derived metabolites are increasingly recognized as markers of disease progression and prognosis [1]. The amine oxide, trimethylamine *N*‐oxide (TMAO), has received particular interest as a potential mediator of cardiovascular (CV) and metabolic disorders [2, 3]. TMAO is produced by hepatic oxidation of trimethylamine, which is generated by gut microbial metabolism of compounds like choline, phosphatidylcholine, and carnitine. Large amounts of free TMAO can also be obtained from seafood sources. TMAO elimination is almost exclusively by renal excretion [4]. Thus, diet, gut microbial composition, and kidney function are important determinants of plasma TMAO levels [5], which show considerable intra‐individual variation [6].

Limited data from mechanistic and animal studies suggest pro‐atherothrombotic and pro‐inflammatory properties of TMAO [1]. Accordingly, recent meta‐analyses of high‐risk patient cohorts reported dose‐dependent associations of baseline TMAO with CV outcomes [7, 8]. However, important inconsistencies exist across observational studies, and the clinical value of circulating TMAO in patient risk stratification is uncertain [5]. Also, data on TMAO and outcomes in the general population are scarce [9].

In the present study, we examined prospective associations of plasma TMAO with long‐term risk of all‐cause, CV, and non‐CV mortality in two large separate cohorts of subjects with different baseline risk: a secondary prevention cohort of patients with suspected chronic coronary syndrome and a primary prevention cohort of community‐dwelling adults.

## Materials and methods

### Study cohorts

Two prospective cohort studies from Western Norway were included. The Western Norway Coronary Angiography Cohort (WECAC) enrolled 4164 patients who underwent elective invasive coronary angiography due to suspected chronic coronary syndrome during 2000–2004 [10]. A total of 61.8% (*n* = 2573) of the patients were additionally included in the Western Norway B‐vitamin Intervention Trial (clinicaltrials.gov:NCT00354081) [11].

The Hordaland Health Study (HUSK) recruited 7051 community‐dwelling adults born during 1925–1927 or 1950–1951, and has been extensively described in previous reports [12]. Baseline examinations were conducted during 1997–1999 (https://husk‐en.w.uib.no/).

Written informed consent was provided by all study subjects. Both study protocols were approved by the Norwegian Data Inspectorate and by the Regional Committee for Medical and Health Research Ethics.

Patients without data on plasma TMAO or covariates included in the multivariable risk models were excluded, leaving 4132 participants in WECAC and 6393 subjects in HUSK for the final analyses.

### Baseline data and biochemical analyses

Previous reports have described the collection of clinical and biochemical data, including the handling and storage of blood samples [10, 13]. Smoking status was defined according to self‐reports and/or plasma cotinine concentrations ≥85 nmol/L. The estimated glomerular filtration rate (eGFR) was calculated using the Chronic Kidney Disease Epidemiology Collaboration formula [14]. In WECAC, angiographically significant coronary artery disease (CAD) was defined by the presence of a lesion with ≥50% stenosis in any main coronary artery (i.e., the left anterior descending artery, the right coronary artery, or the circumflex artery), including their major side branches [15]. Left ventricular ejection fraction (LVEF) was determined by echocardiography or ventriculography.

Plasma TMAO was analyzed by BEVITAL AS (www.bevital.no) using liquid chromatography–tandem mass spectrometry (LC‐MS/MS) [16]. Participants of HUSK and the majority (68.5%) of subjects in WECAC were nonfasting (patients reporting >6 h since their last meal were considered fasting).

### Study endpoints and follow‐up

The endpoints were all‐cause, CV, and non‐CV mortality. CV mortality was classified according to the *10^th^ Revision of the International Classification of Diseases* (codes I00–I99 or R96). Endpoint data were obtained from the Norwegian Cause of Death Registry, using each participant's unique 11‐digit personal identification number. The Norwegian Cause of Death Registry covers more than 98% of the Norwegian population [17]. Study subjects were followed until death or through 2012.

### Statistical analyses

Continuous variables were reported as means (standard deviations [SDs]) and categorical variables as percentages. Baseline concentrations of TMAO were additionally reported as medians (interquartile ranges [IQRs]). Differences in baseline characteristics across TMAO quartiles were assessed using linear regression for continuous variables and logistic regression for categorical variables.

Univariate, age‐, and sex‐adjusted (Model 1) and multivariable (Model 2) Cox regression models were used to obtain hazard ratios (HRs) and corresponding 95% confidence intervals. HRs are reported per one SD increment of log‐transformed plasma TMAO and across TMAO quartiles. Model 2 was adjusted for age (continuous), sex (binary), hypertension (binary), diabetes mellitus (binary), total cholesterol (continuous), smoking (binary), body mass index (continuous), and eGFR (continuous). Potential effect modifications according to variables in Model 2 and LVEF (≤40 vs. >40%) were assessed by adding interaction terms to Model 2. In WECAC, we additionally performed a sensitivity analysis restricted to patients with angiographically significant CAD at baseline.

We used the statistical package SPSS (version 27; SPSS IBM). Reported probability values were two‐tailed, and a *p* < 0.05 was considered statistically significant. Subgroup analyses were considered exploratory and were reported without adjustments for multiple comparisons.

## Results

### Baseline characteristics

Baseline characteristics of the two study cohorts are presented in Table 1. Of the 4132 patients in WECAC, 71.9% were men, 46.6% had hypertension, and 31.7% were current smokers. The mean (SD) age at inclusion was 62 [10] years. A total of 3095 (74.9%) had angiographically significant CAD. Mean (SD) and median (IQR) TMAO concentrations were 9.03 (12.5) and 5.7 (3.6–9.7) μmol/L, respectively.

**Table 1:** Baseline characteristics of patients with suspected coronary heart disease (WECAC) and community‐based subjects (Hordaland Health Study cohort) according to quartiles of plasma trimethylamine N‐oxide

WECAC (*n* = 4132)					
	Quartile 1 (<3.6)	Quartile 2 (3.6–5.7)	Quartile 3 (5.7–9.7)	Quartile 4 (>9.7)	*P*_trend_
Age, years	58 (11)	61 (10)	63 (10)	65 (10)	<0.001
Female sex, %	33.4	29.5	25.8	23.6	<0.001
BMI, kg/m^2^	26 (4)	26 (4)	26 (4)	26 (4)	0.61
Hypertension, %	39.6	44.9	46.8	55.3	<0.001
Diabetes mellitus, %	8.0	10.2	12.9	16.2	<0.001
Current smoking, %	35.5	33.9	31.0	26.3	<0.001
eGFR, ml/min per 1.73 m^2^	95 (13)	90 (14)	86 (16)	80 (20)	<0.001
Total cholesterol, mmol/L	5.1 (1.2)	5.1 (1.1)	5.1 (1.2)	5.0 (1.2)	0.06
LDL‐C, mmol/L	3.1 (1.0)	3.1 (1.0)	3.1 (1.0)	3.1 (1.0)	0.33
HDL‐C, mmol/L	1.3 (0.4)	1.3 (0.4)	1.3 (0.4)	1.3 (0.4)	0.14
Left ventricular ejection fraction, %	65 (10)	64 (11)	63 (12)	63 (12)	<0.001
CRP, mg/L	3.6 (6.9)	3.8 (7.4)	3.7 (7.5)	3.7 (7.0)	0.78
Extent of CAD, %					<0.001
No stenotic vessels	29.9	25.7	23.7	21.0	
One‐vessel disease	24.4	25.2	21.0	22.3	
Two‐vessel disease	20.5	23.8	23.2	21.6	
Three‐vessel disease	25.1	25.2	32.2	35.1	
Prior CVD, %					
AMI	37.0	42.1	40.5	41.4	0.08
PAD	6.0	8.9	9.6	11.9	<0.001
PCI	18.4	18.4	18.2	21.2	0.25
CABG	9.9	11.1	11.8	13.4	0.09
TMAO precursors (plasma)					
Choline, μmol/L	9.2 (2.1)	9.8 (2.4)	10.4 (2.6)	10.8 (3.2)	<0.001
Betaine, μmol/L	39.1 (13.4)	40.3 (12.9)	41.7 (14.7)	42.5 (13.2)	<0.001
Gamma‐butyrobetaine, μmol/L	0.97 (0.23)	1.02 (0.23)	1.07 (0.25)	1.14 (0.31)	<0.001
Carnitine, μmol/L	38.4 (7.4)	39.3 (7.6)	39.6 (7.7)	39.9 (8.2)	<0.001
TML, μmol/L	0.62 (0.25)	0.68 (0.27)	0.76 (0.32)	1.04 (0.73)	<0.001

Hordaland Health Study cohort (*n* = 6393)					
	Quartile 1 (<3.1)	Quartile 2 (3.1–4.7)	Quartile 3 (4.7–8.3)	Quartile 4 (>8.3)	*P*_trend_
Age, years	52 (10)	58 (12)	62 (12)	63 (12)	<0.001
Female sex, %	63.1	57.9	53.4	47.4	<0.001
BMI, kg/m^2^	25 (4)	26 (4)	26 (4)	26 (4)	<0.001
Hypertension, %	9.0	14.9	22.4	26.2	<0.001
Diabetes mellitus, %	1.1	2.5	4.5	6.3	<0.001
Current smoking, %	33.6	29	21.5	17.4	<0.001
eGFR, ml/min per 1.73m^2^	77 (11)	72 (12)	69 (13)	66 (13)	<0.001
Total cholesterol, mmol/L	5.8 (1.0)	6.0 (1.1)	6.0 (1.1)	6.0 (1.1)	<0.001
Plasma TML, μmol/L	0.51 (0.13)	0.55 (0.16)	0.60 (0.17)	0.81 (0.48)	<0.001

*Note*: Continuous variables are presented as means (standard deviation) and categorical variables are reported as percentages.

Abbreviations: AMI, acute myocardial infarction; BMI, body mass index; CABG, coronary artery bypass grafting; CAD, coronary artery disease; CRP, C‐reactive protein; CVD, cardiovascular disease; eGFR, estimated glomerular filtration rate; HDL‐C, high density lipoprotein cholesterol; LDL‐C, low‐density lipoprotein cholesterol; PAD, peripheral artery disease; PCI, percutaneous coronary intervention; TMAO, trimethylamine *N*‐oxide; TML, trimethyllysine; WECAC, Western Norway Coronary Angiography Cohort.

Of the 6393 HUSK participants, 44.5% were men, 18.1% had hypertension, and 25.4% were current smokers. Mean (SD) and median (IQR) TMAO concentrations were 9.1 (16.8) and 4.7 (3.1–8.3) μmol/L, respectively.

Across both cohorts, subjects with higher TMAO concentrations were more likely to have diabetes and hypertension, whereas a lower proportion were smokers. Plasma TMAO showed a negative association with eGFR and LVEF. Positive associations were observed with circulating TMAO precursors such as choline, betaine, trimethyllysine, and gamma‐butyrobetaine.

### TMAO and risk of mortality

The mean (SD) follow‐up time was 9.8 (2.6) and 10.5 (1.9) years in WECAC and HUSK, respectively.

**Fig. 1:**
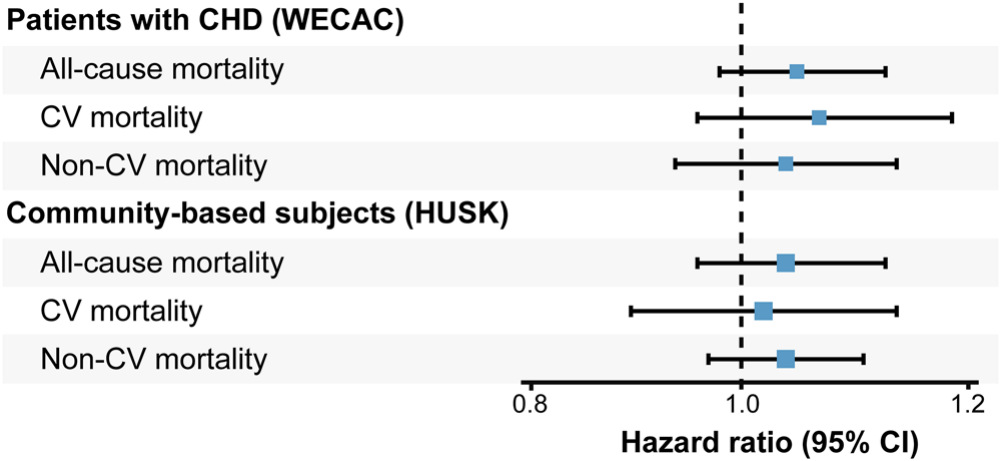
Hazard ratios for mortality per one standard deviation increment of log‐transformed trimethylamine N‐oxide. Adjusted for age, sex, body mass index, diabetes mellitus, smoking, hypertension, estimated glomerular filtration rate, and total cholesterol.

In WECAC, 905 (21.9%) patients died, 413 from CV and 492 from non‐CV causes. In univariate analyses, TMAO was significantly associated with all endpoints. However, these risk relationships were fully attenuated after multivariable adjustments (Table 2). In Model 2, the HRs for all‐cause, CV, and non‐CV mortality per one‐SD increment of log‐transformed TMAO were 1.04 (0.97–1.12), 1.06 (0.95–1.18), and 1.03 (0.93–1.13), respectively (Table 2 and Fig. 1). The risk estimates did not differ according to the fasting status (data not shown).

**Table 2:** Risk association between plasma trimethylamine N‐oxide and mortality in patients with suspected coronary heart disease (Western Norway Coronary Angiography Cohort, n = 4132)

	Unadjusted		Model 1^a^		Model 2^b^	
Plasma TMAO	HR (95% CI)	*P‐*value	HR (95% CI)	*P‐*value	HR (95% CI)	*P‐*value
All‐cause mortality						
Quartiles						
Q1	Reference		Reference		Reference	
Q2	1.33 (1.07–1.65)	0.01	1.05 (0.84–1.30)	0.68	0.98 (0.79–1.22)	0.86
Q3	1.82 (1.48–2.23)	<0.001	1.21 (0.99–1.49)	0.07	1.08 (0.87–1.33)	0.49
Q4	2.29 (1.88–2.79)	<0.001	1.32 (1.08–1.62)	0.01	1.07 (0.86–1.32)	0.56
Trend	1.32 (1.24–1.40)	<0.001	1.10 (1.04–1.18)	0.02	1.03 (0.96–1.10)	0.39
Per one SD^c^	1.34 (1.26–1.42)	<0.001	1.14 (1.07–1.22)	<0.001	1.04 (0.97–1.12)	0.28
CV mortality						
Quartiles						
Q1	Reference		Reference		Reference	
Q2	1.59 (1.13–2.23)	0.007	1.24 (0.88–1.74)	0.22	1.15 (0.82–1.61)	0.42
Q3	2.41 (1.76–3.31)	<0.001	1.57 (1.14–2.17)	0.005	1.36 (0.98–1.88)	0.06
Q4	2.73 (2.00–3.72)	<0.001	1.52 (1.11–2.09)	0.01	1.16 (0.83–1.62)	0.38
Trend	1.38 (1.26–1.51)	<0.001	1.14 (1.04–1.26)	0.005	1.05 (0.95–1.15)	0.38
Per one SD^c^	1.40 (1.28–1.53)	<0.001	1.19 (1.08–1.31)	0.001	1.06 (0.95–1.18)	0.28
Non‐CV mortality						
Quartiles						
Q1	Reference		Reference		Reference	
Q2	1.18 (0.89–1.56)	0.26	0.93 (0.70–1.24)	0.63	0.88 (0.66–1.17)	0.38
Q3	1.45 (1.11–1.90)	0.07	0.99 (0.75–1.30)	0.92	0.90 (0.68–1.19)	0.46
Q4	2.02 (1.57–2.61)	<0.001	1.20 (0.92–1.56)	0.18	1.02 (0.77–1.34)	0.90
Trend	1.27 (1.17–1.38)	<0.001	1.07 (0.99–1.17)	0.09	1.02 (0.93–1.11)	0.70
Per one SD^c^	1.29 (1.18–1.40)	<0.001	1.10 (1.00–1.20)	0.04	1.03 (0.93–1.13)	0.62

Abbreviations: CI, confidence interval; CV, cardiovascular; HR, hazard ratio; Q1, first quartile; Q4, fourth quartile; SD, standard deviation; TMAO, trimethylamine *N*‐oxide.

^a^Adjusted for age and sex.

^b^Adjusted for age and sex, body mass index, diabetes mellitus, smoking, hypertension, estimated glomerular filtration rate, and total cholesterol.

^c^Log transformed.

In HUSK, a total of 884 subjects (13.8%) died, 287 from CV and 597 from non‐CV causes. As in WECAC, TMAO was a strong predictor of mortality in univariate analyses. However, the associations did not persist in models adjusted for age and sex (Table 3). In multivariable adjusted analyses, the HR per one‐SD increment of log‐transformed TMAO was 1.03 (0.96–1.10) for all‐cause mortality, with numerically similar results for CV and non‐CV mortality (all *p* ≥ 0.28).

**Table 3:** Risk associations between plasma trimethylamine N‐oxide and risk of mortality in community‐based subjects (Hordaland Health Study cohort, n = 6393)

	Unadjusted		Model 1^a^		Model 2^b^	
Plasma TMAO	HR (95% CI)	*P‐*value	HR (95% CI)	*P‐*value	HR (95% CI)	*P‐*value
All‐cause mortality						
Quartiles						
Q1	Reference		Reference		Reference	
Q2	1.75 (1.39–2.21)	<0.001	1.01 (0.80–1.28)	0.95	1.03 (0.82–1.31)	0.80
Q3	2.44 (1.95–3.04)	<0.001	1.00 (0.80–1.25)	0.99	1.03 (0.82–1.29)	0.80
Q4	3.07 (2.48–3.80)	<0.001	1.09 (0.88–1.37)	0.43	1.11 (0.88–1.40)	0.38
Trend	1.41 (1.33–1.50)	<0.001	1.03 (0.97–1.10)	0.34	1.03 (0.97–1.11)	0.34
Per one SD^c^	1.35 (1.27–1.42)	<0.001	1.04 (0.97–1.11)	0.29	1.03 (0.96–1.10)	0.49
CV mortality						
Quartiles						
Q1	Reference		Reference		Reference	
Q2	1.55 (1.00–2.38)	0.05	0.81 (0.52–1.25)	0.33	0.79 (0.51–1.23)	0.30
Q3	2.62 (1.76–3.90)	<0.001	0.93 (0.62–1.39)	0.73	0.87 (0.58–1.31)	0.51
Q4	3.75 (2.56–5.49)	<0.001	1.13 (0.77–1.70)	0.54	0.97 (0.65–1.46)	0.88
Trend	1.56 (1.39–1.74)	<0.001	1.09 (0.97–1.12)	0.15	1.03 (0.91–1.17)	0.65
Per one SD^c^	1.43 (1.30–1.57)	<0.001	1.08 (0.97–1.21)	0.18	1.01 (0.89–1.13)	0.94
Non‐CV mortality						
Quartiles						
Q1	Reference		Reference		Reference	
Q2	1.84 (1.40–2.43)	<0.001	1.11 (0.84–1.46)	0.48	1.15 (0.87–1.52)	0.32
Q3	2.36 (1.81–3.07)	<0.001	1.03 (0.78–1.35)	0.83	1.11 (0.84–1.46)	0.46
Q4	2.77 (2.14–3.60)	<0.001	1.07 (0.82–1.40)	0.64	1.17 (0.88–1.54)	0.28
Trend	1.35 (1.26–1.46)	<0.001	1.01 (0.93–1.09)	0.87	1.04 (0.95–1.13)	0.41
Per one SD^c^	1.31 (1.22–1.40)	<0.001	1.01 (0.94–1.10)	0.73	1.03 (0.95–1.12)	0.46

Abbreviations: CI, confidence interval; CV, cardiovascular; HR, hazard ratio; Q1, first quartile; Q4, fourth quartile; SD, standard deviation; TMAO, trimethylamine *N*‐oxide.

^a^Adjusted for age and sex.

^b^Adjusted for age, sex, body mass index, diabetes mellitus, smoking, hypertension, estimated glomerular filtration rate, and total cholesterol.

^c^Log transformed.

Analyses comparing the fourth versus first TMAO quartile or trends across quartiles yielded comparable results across both cohorts (Tables 2 and 3).

### Sensitivity and subgroup analyses

A sensitivity analysis restricted to patients with angiographically significant CAD in WECAC (*n* = 3095) showed a similar null association between plasma TMAO and mortality (Table S1).

Subgroup analyses according to baseline characteristics are shown in Table S2. The risk estimates were generally consistent in subgroups, including sex, prevalent hypertension, and diabetes. Notably, across both cohorts, there was a more pronounced TMAO–mortality relationship in patients with lower eGFR. However, the differences were numerically small and not statistically significant in WECAC. There was no interaction according to baseline LVEF (available in WECAC only).

To examine the importance of eGFR as a potential confounder in WECAC, we additionally examined risk associations of TMAO with mortality in Model 2 without inclusion of eGFR as a covariate. In this simplified model, the HRs per one‐SD increment of log‐transformed TMAO were 1.12 (1.05–1.20), 1.15 (1.04–1.27), and 1.09 (1.00–1.20) for all‐cause, CV, and non‐CV mortality, respectively.

## Discussion

### Principal findings

In two large separate cohorts of patients with suspected coronary heart disease (CHD) or community‐based adults, plasma TMAO was not predictive of long‐term (∼10 years) all‐cause, CV, or non‐CV mortality. These findings were consistent in subgroups of patients with angiographically verified CAD and subjects with reduced LVEF.

### TMAO and clinical outcomes

In recent meta‐analyses—which primarily include high‐risk patient populations with established CV disease (CVD) or chronic kidney disease—elevated TMAO was related to the risk of CV events [7] and mortality [18]. In a community‐based case‐control study of older adults [19], TMAO was associated with incident atherosclerotic CVD. On the other hand, no relationship of TMAO with CV events was observed among subjects free of CVD in a case‐cohort study from the PREDIMED trial during a 4.8‐year follow‐up [20]. Similar null associations were recently reported in diabetic patients [21, 22]. Observational studies among patients with established CVD also show inconsistencies [5]. For instance, prospective studies on CV patients reported no independent associations of TMAO with acute myocardial infarction [23] or incident CVD events [24].

Consistent across the primary and secondary prevention cohorts of the present study, TMAO was not predictive of all‐cause, CV, or non‐CV mortality after multivariable adjustments, suggesting that TMAO does not improve risk prediction beyond the established risk factors. In contrast to a recent meta‐analysis [25] and a multicenter European study [26], we found similar results in patients with verified CAD and patients with reduced LVEF. However, only a small subset of WECAC patients had LVEF ≤40%, necessitating cautious interpretation of this subgroup analysis.

Geographic, ethnic, and dietary variations [5, 27, 28]—as well as differences in follow‐up, endpoint definitions, and model adjustments—may underlie discrepancies across studies evaluating TMAO as a marker of adverse prognosis. As evident for other emerging biomarkers [29], the magnitude and validity of the reported risk associations may also be subject to publication bias [18]. Of note, the size of the population sample of the present study was higher than one fourth of the largest meta‐analysis on TMAO and mortality published to date [8].

### TMAO and renal function

TMAO is primarily eliminated unchanged in urine and is markedly elevated in patients with chronic kidney disease [30]. Accordingly, we observed negative associations of plasma TMAO with eGFR in both cohorts. Based on limited data from animal models [30, 31, 32] showing renal damage from elevated TMAO concentrations, a potential bidirectional relationship of plasma TMAO and kidney disease has been hypothesized. In contrast, a recent Mendelian randomization analysis found no association of genetically elevated TMAO with renal dysfunction or cardiometabolic health [33].

In line with some [34, 35]—but not all [36]—previous investigations, there was a trend towards a stronger relationship of TMAO with mortality in patients with lower eGFR. However, the differences were numerically small and not statistically significant across the cohorts. Of note, TMAO was predictive of mortality in multivariable models in WECAC after exclusion of eGFR as a covariate. Indeed, impaired renal function has been suggested as a major confounder in analyses of TMAO‐related adverse outcomes in patients with established CHD [24, 37], and our results clearly support that eGFR attenuates the associations of TMAO with adverse outcomes.

### TMAO and non‐CV mortality

Recent observational data have suggested relationships of TMAO with leading causes of non‐CV mortality, such as dementia [38, 39] and carcinogenesis [40]. However, a potential causal role of TMAO is largely unexplored. To our knowledge, this is the first study to assess associations of plasma TMAO with non‐CV mortality, which was the main contributor to all‐cause mortality both in the general population sample and for patients with CHD. As observed for CV mortality, TMAO had no predictive value for non‐CV mortality in either study cohort.

### Strengths and limitations

Major strengths of this prospective study include the large number of participants, the long‐term follow‐up and the replication of results in two separate patient populations with different baseline risks. Further, endpoint data were obtained from a national registry with almost 100 percent coverage. Approximately 45 percent of the total study population were women, providing adequate gender representation. Another strength is that samples were analyzed simultaneously at the same laboratory by technicians blinded to clinical data, and TMAO and precursors were measured by a targeted LC‐MS/MS method that included authentic labelled internal standards for all metabolites, thereby providing high precision and accuracy and no assay interference [41].

We are aware of several limitations. First, we included only single baseline TMAO measurements in the analyses, which may result in underestimation of true risk relationships by regression dilution bias, due to low within‐subject reproducibility of TMAO [42]. Second, study participants were mainly Caucasian subjects from Western Norway, potentially limiting generalizability to other populations. Third, the majority of participants provided nonfasting blood samples. Fourth, inherent to any observational design, inferences of causation cannot be drawn, although the clinical utility of a biomarker for risk stratification does not necessarily depend on causal relationships [43].

## Conclusions

In this prospective study of more than 10,000 subjects with or without established CHD, circulating TMAO did not predict long‐term risk of all‐cause, CV, or non‐CV mortality. Our results do not support the use of TMAO for patient risk stratification in primary or secondary prevention.

## Funding

This research received no specific grant from any funding agency in the public, commercial, or not‐for‐profit sectors.

## Conflicts of interest

The authors declare that there are no conflicts of interest that could be perceived as prejudicing the impartiality of the research reported.

## Author contributions

Espen Ø. Bjørnestad: Conceptualization; Formal analysis; Methodology; Visualization; Writing ‐ original draft; Writing ‐ review and editing. Indu Dhar: Data curation; Formal analysis; Methodology; Visualization; Writing – original draft; Writing – review and editing. Gard F. T. Svingen: Investigation; Methodology; Writing ‐ review and editing. Eva R. Pedersen: Investigation; Methodology; Writing ‐ review and editing. Stein Ørn: Investigation; Supervision; Writing – review and editing. Mads M. Svenningsson: Investigation; Writing – review and editing. Grethe S. Tell: Investigation; Methodology; Project administration; Writing ‐ review and editing. Per M. Ueland: Investigation; Methodology; Project administration; Writing ‐ review and editing. Gerhard Sulo: Formal analysis; Methodology; Writing – review and editing. Reijo Laaksonen: Investigation; Methodology; Writing – review and editing. Ottar Nygård: Conceptualization; Formal analysis; Investigation; Methodology; Project administration; Supervision; Writing ‐ review and editing.

## Supporting information


**Supplemental Table 1**: Risk‐associations between plasma trimethylamine *N*‐oxide and mortality in patients (*n* = 3095) with angiographically significant coronary artery disease.
**Supplemental Table 2**: Risk‐associations between plasma trimethylamine N‐oxide (log transformed) and mortality according to subgroups.
