# Supplementary material for: Circulating trimethylamine N‐oxide levels do not predict 10‐year survival in patients with or without coronary heart disease
Source: J Intern Med. 2022 Aug 9;292(6):915–24. doi: 10.1111/joim.13550 (PMC9804190; doi:10.1111/joim.13550)
Supplement: Supplementary file 1 — Supplemental Table 1: Risk‐associations between plasma trimethylamine N‐oxide and mortality in patients (n = 3095) with angiographically significant coronary artery disease. Supplemental Table 2: Risk‐associations between plasma trimethylamine N‐oxide (log transformed) and mortality according to subgroups. [file JOIM-292-915-s001.docx]

**Supplemental material**

**Circulating trimethylamine *N*-oxide levels do not predict 10-year survival in patients with or without coronary heart disease**

Espen Ø. Bjørnestad^1^, MD; Indu Dhar^2^, PhD; Gard FT Svingen^3^, MD, PhD; Eva R

Pedersen^3,4^, MD, PhD; Stein Ørn^1^, MD, PhD; Mads M Svenningsson, MD^3^; Grethe S Tell, MPH^5^, PhD; Per M Ueland^4^, MD, PhD; Gerhard Sulo^6^, MD, PhD; Reijo Laaksonen^7^, MD, PhD; Ottar Nygård^3,4^, MD, PhD.

*^1^Department of Cardiology, Stavanger University Hospital, Stavanger, Norway*

*^2^Mohn Nutrition Research Laboratory, Department of Clinical Science, University of Bergen,*

*Bergen, Norway*

*^3^Department of Cardiology, Haukeland University Hospital, Bergen, Norway*

*^4^Department of Clinical Science, University of Bergen, Bergen, Norway*

*^5^Department of Global Public Health and Primary Care, University of Bergen, Bergen,*

*Norway*

*^6^Centre for Disease Burden, Norwegian Institute of Public Health, Bergen, Norway*

*^7^Finnish Cardiovascular Research Center, University of Tampere, Tampere, Finland*

| **Supplemental Table 1. Risk-associations between plasma trimethylamine *N*-oxide and mortality in patients (n=3095) with angiographically significant coronary artery disease** | | | | |
| --- | --- | --- | --- | --- |
| **All-Cause Mortality** | |  | Model 2^a^ | |
|  |  |  | HR (95% CI) | *P-*Value |
| Plasma TMAO | |  |  |  |
|  | Quartiles |  |  |  |
|  | Q1 |  | Reference |  |
|  | Q2 |  | 0.91 (0.72-1.16) | 0.45 |
|  | Q3 |  | 0.99 (0.79-1.24) | 0.93 |
|  | Q4 |  | 0.98 (0.78-1.23) | 0.85 |
| **CV Mortality** | |  | | |
|  | Quartiles |  |  |  |
|  | Q1 |  | Reference |  |
|  | Q2 |  | 1.05 (0.74-1.51) | 0.77 |
|  | Q3 |  | 1.22 (0.87-1.71) | 0.26 |
|  | Q4 |  | 1.09 (0.77-1.55) | 0.64 |
| **Non-CV Mortality** | |  | | |
|  | Quartiles |  |  |  |
|  | Q1 |  | Reference |  |
|  | Q2 |  | 0.82 (0.60-1.13) | 0.22 |
|  | Q3 |  | 0.83 (0.61-1.13) | 0.24 |
|  | Q4 |  | 0.91 (0.67-1.12) | 0.53 |
| ^a^Adjusted for age, sex, BMI, diabetes mellitus, smoking, hypertension, eGFR, and total cholesterol | | | | |

| **Supplemental Table 2. Risk-associations between plasma trimethylamine N-oxide (log transformed) and mortality according to subgroups** | | | | | | | | | |
| --- | --- | --- | --- | --- | --- | --- | --- | --- | --- |
|  | |  | WECAC | | |  | HUSK | | |
|  | |  | HR (95% CI) * |  | P_int_ |  | HR (95% CI) * |  | P_int_ |
| Age | |  |  |  |  |  |  |  |  |
|  | ≤ median |  | 1.05 (0.90-1.22) |  | 0.39 |  | 1.15 (0.92-1.43) |  | 0.43 |
|  | > median |  | 1.04 (0.96-1.13) |  |  |  | 1.01 (0.94-1.09) |  |  |
| Sex | |  |  |  |  |  |  |  |  |
|  | Females |  | 1.05 (0.90-1.22) |  | 0.80 |  | 1.05 (0.94-1.16) |  | 0.78 |
|  | Males |  | 1.04 (0.96-1.13) |  |  |  | 1.01 (0.92-1.11) |  |  |
| BMI | |  |  |  |  |  |  |  |  |
|  | ≤ median |  | 1.01 ( 0.92-1.11) |  | 0.47 |  | 1.04 (0.95-1.15) |  | 0.98 |
|  | > median |  | 1.08 (0.97-1.20) |  |  |  | 1.00 (0.91-1.10) |  |  |
| Hypertension | |  |  |  |  |  |  |  |  |
|  | No |  | 1.06 (0.95-1.19) |  | 0.24 |  | 1.03 (0.95-1.12) |  | 0.64 |
|  | Yes |  | 1.03 (0.94-1.13) |  |  |  | 1.01 (0.90-1.14) |  |  |
| Diabetes | |  |  |  |  |  |  |  |  |
|  | No |  | 1.05 (0.97-1.14) |  | 0.27 |  | 1.04 (0.96-1.12) |  | 0.74 |
|  | Yes |  | 0.99 (0.84-1.17) |  |  |  | 0.91 (0.75-1.11) |  |  |
| Smoking | |  |  |  |  |  |  |  |  |
|  | No |  | 1.07 (0.98-1.17) |  | 0.03 |  | 1.03 (0.95-1.11) |  | 0.85 |
|  | Yes |  | 0.98 (0.86-1.11) |  |  |  | 1.02 (0.89-1.18) |  |  |
| Total cholesterol | | | |  |  |  |  |  |  |
|  | ≤ median |  | 1.07 (0.97-1.19) |  | 0.32 |  | 0.99 (0.89-1.09) |  | 0.41 |
|  | > median |  | 1.01 (0.91-1.12) |  |  |  | 1.05 (0.96-1.16) |  |  |
| eGFR | | | |  |  |  |  |  |  |
|  | ≤ median |  | 1.14 (1.05-1.23) |  | 0.13 |  | 1.17 (1.02-1.34) |  | 0.04 |
|  | > median |  | 1.01 (0.88-1-16) |  |  |  | 0.91 (0.98-1.07) |  |  |
| LVEF | | | |  |  |  |  |  |  |
|  | ≤40 |  | 1.11 (0.89-1.38) |  | 0.11 |  | N/A |  |  |
|  | >40 |  | 1.02 (0.94-1.10) |  |  |  | N/A |  |  |
| BMI, body mass index; CI, confidence interval; HR, hazard ratio; LVEF, Left ventricular ejection fraction; WECAC, Western Norway Coronary Angiography Cohort. | | | | | | | | | |
| * Per 1 SD. Adjusted for age and sex, BMI, diabetes mellitus, smoking, hypertension, eGFR, and total cholesterol | | | | | | | | | |
